# Gynecological Cancers and Microbiota Dynamics: Insights into Pathogenesis and Therapy

**DOI:** 10.3390/ijms25042237

**Published:** 2024-02-13

**Authors:** Giovanna Cocomazzi, Lino Del Pup, Viviana Contu, Gabriele Maggio, Lodovico Parmegiani, Walter Ciampaglia, Daniele De Ruvo, Raffaele Faioli, Annamaria Maglione, Giorgio Maria Baldini, Domenico Baldini, Valerio Pazienza

**Affiliations:** 1 Division of Gastroenterology, Fondazione IRCCS-Casa Sollievo della Sofferenza, 71013 San Giovanni Rotondo, FG, Italy; g.cocomazzi@operapadrepio.it; 2 Gynecological Endocrinology and Fertility, University Sanitary Agency Friuli Central (ASUFC), Via Pozzuolo, 330, 33100 Udine, FVG, Italy; info@delpupginecologia.it; 3 Integrative Medicine Unit, Humanitas Gradenigo, Corso Regina Margherita 8/10, 10153 Torino, FC, Italy; viviana.contu@gradenigo.it; 4 Pia Fondazione Cardinale Giovanni Panico, Via S. Pio X, 4, 73039 Tricase, LE, Italy; gabrielemaggio.gm@alice.it; 5 Next Fertility GynePro, NextClinics International Via T. Cremona 8, 40137 Bologna, RE, Italy; lodovico.parmegiani@gynepro.it (L.P.); walter.ciampaglia@gynepro.it (W.C.); 6 Gynaecology, Obstetrics and Reproductive Medicine Affidea Promea, Via Menabrea 14, 10126 Torino, TO, Italy; daniele.deruvo@gmail.com; 7 Gynecology and Obstetrics, IRCCS “Casa Sollievo della Sofferenza”, 71013 San Giovanni Rotondo, FG, Italy; raffaelefaioli@hotmail.it (R.F.); a.maglione@operapadrepio.it (A.M.); 8 IVF Center, Momò Fertilife, 76011 Bisceglie Via Cala dell’Arciprete, 76011 Bisceglie, BT, Italy; gbaldini97@gmail.com (G.M.B.); dbaldini@libero.it (D.B.)

**Keywords:** microbiota, gynecological cancer, cervical cancer, ovarian cancer, endometrial cancer, estrobolome

## Abstract

In recent years, the relationship between the microbiota and various aspects of health has become a focal point of scientific investigation. Although the most studied microbiota concern the gastrointestinal tract, recently, the interest has also been extended to other body districts. Female genital tract dysbiosis and its possible impact on pathologies such as endometriosis, polycystic ovary syndrome (PCOS), pelvic inflammatory disease (PID), and gynecological cancers have been unveiled. The incursion of pathogenic microbes alters the ecological equilibrium of the vagina, triggering inflammation and compromising immune defense, potentially fostering an environment conducive to cancer development. The most common types of gynecological cancer include cervical, endometrial, and ovarian cancer, which occur in women of any age but especially in postmenopausal women. Several studies highlighted that a low presence of lactobacilli at the vaginal level, and consequently, in related areas (such as the endometrium and ovary), correlates with a higher risk of gynecological pathology and likely contributes to increased incidence and worse prognosis of gynecological cancers. The complex interplay between microbial communities and the development, progression, and treatment of gynecologic malignancies is a burgeoning field not yet fully understood. The intricate crosstalk between the gut microbiota and systemic inflammation introduces a new dimension to our understanding of gynecologic cancers. The objective of this review is to focus attention on the association between vaginal microbiota and gynecological malignancies and provide detailed knowledge for future diagnostic and therapeutic strategies.

## 1. Introduction

The female reproductive tract is characterized by a unique microbiota that accounts for approximately 9% of the body’s total microbial population [1]. The healthy vaginal microbiota is dominated by the *Lactobacillus* genus, including *Lactobacillus crispatus*, *Lactobacillus iners*, *Lactobacillus jensenii*, and *Lactobacillus gasseri* [2]. Lactobacilli are involved in maintaining the typical vaginal ecosystem by preventing the excessive growth of pathogenic and opportunistic microorganisms through competition for nutrients, adherence to the vaginal epithelium, modulation of the local immune system, and reduction in vaginal pH-producing lactic acid and antimicrobial metabolites, such as bacteriocins and hydrogen peroxide (H_2_O_2_) [3]. Ravel et al. [1] proposed a classification of vaginal microbiota into five main types termed “Community State Type” (CST) differentiated as CST I, CST II, CST III, and CST V, all dominated by different and specific *Lactobacillus* spp., while the CST IV is further divided into the two sub-types CST IV-A and CST IV-B (Gajer et al., 2012) [4]. CST IV-A is dominated by various species of anaerobic bacteria such as *Anaerococcus*, *Finegoldia*, *Corynebacterium*, and some genera of *Streptococcus*, while CST IV-B is dominated by a higher proportion of the genus *Atopobium* together with *Prevotella*, *Parvimonas*, *Gardnerella*, *Peptoniphilus*, *Sneathia*, or *Mobiluncus* [4].

Microbial communities colonizing the human vagina undergo changes in species type and abundance due to several factors that may include age, puberty and sexual activity, menopause, hormonal fluctuations, medication use, and intimate hygiene [5,6]. The preservation of a high number of resident lactobacilli is an effective hallmark of a woman’s health condition. Conversely, an abnormal vaginal microbiota that leads to a strong reduction or disappearance of lactobacilli is at the basis of the development of bacterial vaginosis (BV) [7]. Alterations in the vaginal microbiota have also been associated with obstetric complications such as preterm birth or late miscarriage and with frequent diseases of the female reproductive tract, namely recurrent urinary tract infections, high risk of contracting sexually transmitted diseases (HIV, HPV, and HSV-2), endometriosis, pelvic inflammatory disease (PID), polycystic ovary syndrome (PCOS), infertility and gynecologic cancers [8,9,10,11,12,13,14,15]. Pathogenic bacteria can modulate the carcinogenesis process through the production of specific toxins, such as cytolethal distending toxins (CDTs) and colibactin, which can damage host DNA or trigger a state of inflammation [16]. Dysbiosis can result in epithelial barrier disruption, immune dysregulation, genotoxicity, and inflammation, contributing to the etiology, disease severity, and response to the clinical management of gynecologic malignancies [17]. A compelling aspect lies in the exploration of the microbiota’s role in modulating the immune system. Evidence suggests that a balanced and diverse microbiota may contribute to a robust immune response, potentially influencing the body’s ability to combat gynecologic cancers [18]. Conversely, dysbiosis, an imbalance in microbial communities, has been associated with increased inflammation and a compromised immune defense, potentially fostering an environment conducive to cancer development.

The intricate relationship between the gut microbiota and systemic inflammation introduces a new dimension to our understanding of gynecologic cancers. The interaction between microbial communities and host cells may affect the tumor microenvironment, influencing cancer cell behavior and response to therapies. Furthermore, emerging research explores the potential role of the vaginal microbiota, specifically in gynecologic health. Variations in the vaginal microbiome have been linked to conditions such as bacterial vaginosis, which, in turn, may influence the susceptibility to certain gynecologic cancers. Understanding these connections could lead to innovative preventive and new therapeutic strategies. Despite the promising strides in this field, it is essential to acknowledge the complexity and nuances of microbiota–gynecologic cancer interactions. The microbiota is highly individualized, and numerous factors, including genetics, lifestyle, and environmental exposures, contribute to its composition. Consequently, translating these findings into personalized clinical interventions poses both challenges and opportunities.

## 2. Gynecologic Cancer and Microbiota

Gynecologic cancers are malignant pathologies that originate in a woman’s reproductive system. An estimated 113,000 women will be diagnosed with gynecologic cancer, and more than 33,000 of them will not survive. The main tumor forms are uterine cancer (endometrium and cervix) and ovarian cancer. Several risk factors can predispose to the onset of gynecologic cancers but generally include a high prevalence of sexually transmitted diseases (HPV, *C. trachomatis*, and HIV infection), hormone therapy use after menopause, infertility, or no history of pregnancy, smoking, alcohol consumption, obesity, reduced physical activity, hormonal imbalances, genetic and epigenetic factors [19,20,21,22,23,24,25]. Although human studies are currently unable to distinguish whether changes in the microbiota are the cause or effect of the tumor, several results demonstrate that the microbiota can promote carcinogenesis, preventing apoptosis or stimulating proliferation and genomic instability [26].

The pathogenic bacteria promote the disruption of the epithelial barrier through hydrolytic enzymes (e.g., sialidase and prolidase) and the release of inflammatory chemokines and cytokines like interleukin (IL)-6, IL-8, or tumor necrosis factor (TNF), reactive oxygen species and other carcinogenic metabolites leading to chronic inflammation and a dysregulated local metabolism [27]. Furthermore, they also lead to genomic instability, directly damaging DNA or inhibiting DNA repair mechanisms, increasing susceptibility to mutations, and promoting the activation of cyclooxygenase 2 and nuclear factor κB (NF-κB,) which inhibit apoptosis and promote angiogenesis [28]. In particular, the production of cytokines such as TNFα, IL-1, IL-6, and IL-17, and the activation of toll-like receptors (TLRs) by pathogenic bacteria produces the activation of NF-κB cascade, a transcription factor of antiapoptotic genes (Bcl-2 and Bcl-xL) causing the increase in cell proliferation and angiogenesis processes [29]. Furthermore, the metabolites derived from gut microbiota entering circulation facilitates the development of distant cancer [30]. For example, the cell wall components such as lipoteichoic acid (LTA) and deoxycholic acid (DCA), a metabolite of Gram-positive intestinal bacteria, have been shown to promote the development of hepatocellular carcinomas via enterohepatic circulation [30,31,32].

A large number of pathogenic bacteria are known to promote cancer; for instance, *Helicobacter pylori* is a causal factor of gastric cancer, and *S. bovis* and *Fusobacterium nucleatum* are associated with colon cancer [33,34,35,36]. Studies in mouse models treated with antibiotics and, therefore, with a depleted microbiota have shown a substantial reduction in the number of tumors in the colon and the liver [37]. Furthermore, the transplantation of fecal material of patients affected by colorectal cancer to germ-free mice caused lesions, epigenetic changes, and DNA alterations [38]. This provides strong evidence of tumor-promoting effects of the dysbiotic intestinal microbiota in different neoplasms [39]. Likewise, evidence supports that modifications of the microbiota of the female reproductive tract influence the development and evolution of gynecologic cancers. The resident microorganisms guarantee protection from invasion by pathogenic microorganisms through the production of antimicrobial and anti-inflammatory factors, while the host provides nutrients [40]. The bacteria that are part of the common vaginal flora establish a symbiosis or, rather, a mutualistic relationship with the human host. On the contrary, some microorganisms that are not part of the resident vaginal microbial flora can cause various pathologies. Thus far, it is not yet entirely clear whether gynecological cancer is due to the presence of single pathogenic bacterial species or is related to global changes in the microbiota, which, in the presence of other host risk factors, can induce tumor development [18]. It is interesting to note that certain bacteria, such as the sexually transmitted pathogen *C. trachomatis*, induce the epithelial–mesenchymal transition of infected cells, promoting tumorigenesis through the loss of epithelial cell adhesion and the downregulation of DNA damage responses [41]. Moreover, the gut–vagina microbiota axis can influence the levels of estrogen, promoting the onset of estrogen-dependent pathologies such as endometriosis and cancer [42]. Also, the gut microbiota regulates circulating estrogen levels; the complex of genes encoding estrogen-metabolizing enzymes is defined as the “estrobolome”, which regulates estrogen through the release of β-glucuronidase—an enzyme that deconjugates estrogens into their active forms—leading to reabsorption and recirculation in the blood, ultimately affecting systemic estrogen levels [42]. Circulating estrogens reach the cells of the vaginal epithelium, which produce glycogen which in turn is used by lactobacilli to produce lactic acid [42]. Consequently, if dysbiosis occurs, the activity of beta-glucuronidase can be altered, causing a deficit or excess of free estrogen.

## 3. Microbiota and Cervical Cancer

**Figure 1:**
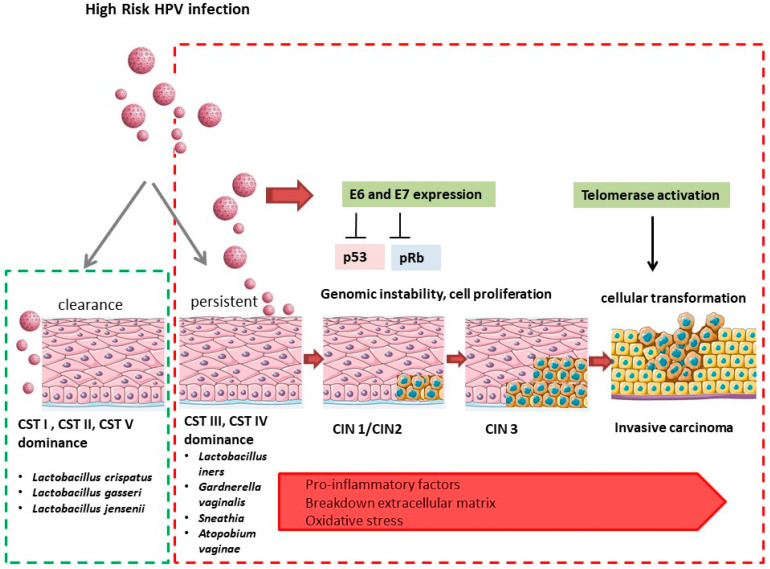
CST I, CST II, and CST V, respectively dominated by *L.crispatus*, *L. gasseri*, and *L. jensenii*, are associated with a rapid clearance of an acute HPV infection. Conversely, in bacterial vaginosis, it assists the depletion of *Lactobacillus* spp., and CST IV and CST III dominance promoting proinflammatory environment, uncontrolled transcription of E6 and E7, genomic instability, viral integration, and telomerase activation, which are crucial for carcinogenesis processes.

According to the World Health Organization, cervical cancer is the fourth most frequent cancer in women, with an estimated 342,000 deaths in 2020. More than 95% of cervical cancer is due to the infection with human papillomavirus (HPV) [43]. HPV infection is very frequent in the population; it is estimated that over 70% of sexually active women become infected during their lifetime, with a peak prevalence in young women up to 25 years of age [44,45]. Cervical cancer has two main histological subtypes; the most common is squamous-cell carcinoma (SCC), which accounts for approximately 70% of all cervical cancers, and adenocarcinoma (ADC), which comprises approximately 20% of all cervical cancers [46]. Several factors have been suggested to increase the risk of cancer development, including promiscuity, tobacco smoke, immunosuppression, long-term oral contraceptive use, sexually transmitted disease, and co-infection with type 2 herpes simplex virus (HSV-2) and human immunodeficiency virus (HIV) [47,48]. There are about 200 HPV virus genotypes that have been identified and classified into low- and high-risk genotypes (LR-HPV and HR-HPV) [49]. Molecular and epidemiological studies have highlighted that there are some strains of HPV most implicated in the formation of CIN (cervical intraepithelial neoplasia) cervical lesions. High-risk HPV genotypes are HPV-16 or HPV-18 [49]. The squamo-columnar junction (SCJ) is mainly susceptible to HPV infection and constitutes the site where tumors develop [50]. The large majority of infections are transient and asymptomatic. However, if the infection persists, it can progress to a productive infection (productive cervical intraepithelial neoplasia (CIN), mainly representing CIN1 and a subset of CIN2) and a transforming infection (CIN2 and CIN3) [51]. The virus interacts with the host cell through its viral factors; in particular, the E6 oncoprotein of human papillomavirus (HPV) types 16 and 18 alters cell growth by binding to the p53 tumor-suppression protein [52]. The E6 protein stimulates the degradation of the p53 protein via ubiquitin-dependent proteolysis [53]. These interactions primarily result in chromosomal instability and the alteration in cellular mechanisms controlling cell growth. The inhibition of these control mechanisms leads to the process of cellular transformation. The HPV E7 oncoprotein interacts with the retinoblastoma protein pRb enhanced phosphorylation and degradation. pRb is a negative regulator of the cell cycle and typically prevents entry into the S phase by associating with the transcription factor E2F [54]. In this case, however, E7 binds to pRb, displacing E2F and beginning the expression of the proteins necessary for DNA replication and promoting cell proliferation [52] (Figure 1). The natural history of the infection is strongly conditioned by the balance that is established between the host and the infecting agent. In fact, there are three possibilities for the evolution of HPV infection: clearance, persistence, and progression [55]. However, 85–90% of high-risk HPV infections resolve spontaneously, and 10–15% can persist, leading to the development of precancerous cervical intraepithelial neoplasia (CIN) and invasive cervical cancer (CCI) [18,19,20,21,22,23,24,25,26,27,28,29,30,31,32,33,34,35,36,37,38,39,40,41,42,43,44,45,46,47,48,49,50,51]. The time between the infection and the onset of precancerous lesions is approximately five years, while the latency for the development of cervical cancer can be decades [55]. HPV infection can cause benign lesions or malignant lesions. The benign lesions induced by HPV include non-genital and anogenital skin and mucous warts and oral and laryngeal papillomas [56]. Malignant tumors that can evolve from persistent HPV infections may include, in addition to cervical cancer, anal cancer and cancers of the penis, vulva, and vagina, as well as some carcinomas of the oral cavity [57]. Emerging evidence suggests that the vaginal microbiota plays a role in cervical carcinogenesis and in the clearance or persistence of HPV. Epidemiological studies have revealed associations between diverse vaginal microbiota not dominated by *Lactobacillus* spp., bacterial vaginosis (BV), and HPV infection and persistence [57,58,59] (Figure 1). Furthermore, recent studies have identified that the abundance of the vaginal species *L. gasseri* is associated with HPV clearance, while *Atopobium* spp. is associated with HPV persistence [60]. Furthermore, it has been observed that the microbiota of women with cervical dysplasia and cervical cancer shows a depletion of *Lactobacillus* spp., compared to healthy individuals and in particular some microorganisms associated with bacterial vaginosis such as *Gardnerella*, *Megasphaera*, *Prevotella*, *Peptostreptococcus*, *Streptococcus*, *Sneathia sanguinegens*, and *Atopobium* are considerably more abundant in the vaginal microbiota of patients with HPV infections [17]. Furthermore, it has been observed that women with a vaginal microbiota dominated by *L. iners* have a higher probability of acquiring a high-risk HPV infection and developing a malignancy as compared to women with a vaginal microbiota dominated by *L. crispatus* [61]. *L. iners* is less able to inhibit the colonization of pathogens and this seems to depend on the production of only the L-lactic acid isoform, which is less protective than the D-lactic acid isoform [62,63]. In addition, the presence of *L. iners* is associated with the presence of several pro-inflammatory cytokines and with the production of inerolysin, a pore-forming cytotoxin similar to the vaginolysin protein produced by *Gardnerella* spp., which favors infections through the formation of pores in the vaginal epithelium [64].

Vaginal dysbiosis can produce a pro-inflammatory environment that increases the persistence of HPV infection and viral transformation, including E6 and E7 expression, genomic instability, and telomerase activation, which causes cellular transformation [65]. The women with vaginal microbiota dominated by CST IV show several pro-inflammatory factors such as TNF-α, IL-1α, IFN-γ, IL-1β, IL-4, IL-12p70, IL-10, and IL-8. Recent studies suggested that the *C. trachomatis* infection is associated with HPV infections and could increase the risk of persistence and progression to high-grade cervical intraepithelial neoplasia (CIN). Although there is no evidence that *C. trachomatis* directly affects the host DNA or the transcription of HPV genes, a number of biological mechanisms with which *C. trachomatis* may increase the risk of cervical cancer have been reported in several studies. It has been observed that *C. trachomatis* disrupts N-Cadherin-dependent cell–cell junctions through the involvement of Ca2+ ions and a reorganization of the actin cytoskeleton and thus increases the exposure of basal cells to HPV [66,67]. *C. trachomatis* may have antiapoptotic effects; the antiapoptotic activity could depend on encoded protein factors that interrupt many different host cell apoptotic pathways [68]. Furthermore, it has been observed that *C. trachomatis* can increase the risk of infection with HPV and its persistence through inhibition of the expression of the γ-inducible interferon of the major complex histocompatibility class II (MHC-II) [69]. The collective action of these conditions could directly influence the development of precancerous lesions and progression to cancer.

## 4. Microbiota and Endometrial Cancer

Endometrial cancer is the most common gynecological cancer in developed countries, and it is the 6th most common cancer in women and the 15th most common cancer worldwide [70]. It mostly occurs in postmenopausal women in their sixth and seventh decades of life [23].

At the base of endometrial cancer, there are genetic and hereditary factors [24]. Other factors, such as socioeconomic status, ethnicity, and environmental factors, including obesity, inflammation, imbalances in estrogen metabolism, and estrogen therapy after menopause, could place some women at a greater risk of developing endometrial cancer [18,19,20,21,22,23,24,25,26,27,28,29,30,31,32,33,34,35,36,37,38,39,40,41,42,43,44,45,46,47,48,49,50,51,52,53,54,55,56,57,58,59,60,61,62,63,64,65,66,67,68,69,70,71,72]. The greater incidence in industrialized countries leads us to hypothesize that environmental and dietary factors, such as a diet rich in fats, may favor an increase in the risk of endometrial carcinoma [73]. Many epidemiological studies have highlighted that obesity can promote the development of endometrial cancer through different mechanisms. These mechanisms include hyperinsulinemia, IGF (insulin-like growth factor), and estrogen. In fact, chronic hyperinsulinemia decreases the concentration of IGF-binding protein 1 (IGFB1) and IGF-binding protein 2 (IGFB2), which increase the bioavailability of free IGF-1, with a concomitant change in the cellular environment (mitogenesis and antiapoptosis. Furthermore, increased estradiol not only increases endometrial cell proliferation and inhibits apoptosis, but it can also stimulate local synthesis of IGF-1 in endometrial tissue [74].

Moreover, chronic hyperinsulinemia can promote tumorigenesis in estrogen-sensitive tissues since it reduces the concentration of sex hormone-binding globulin (SHBG) in the blood and increases the bioavailability of estrogens [74]. Several published studies have highlighted how estrogen therapy attenuates menopause symptoms and improves women’s quality of life, increasing the risk of endometrial cancer [74].

The increase in the incidence of endometrial cancer, with the increase in obesity in postmenopausal women, depends on endometrial proliferation caused by endogenous estradiol production by adipose tissue. Estradiol increases in parallel with BMI in postmenopausal women. For this reason, menopausal women are currently treated not only with estrogens but also with progestins that hinder the proliferative effects of estrogens on the endometrium [75]. According to recent studies, the microbiota may also be an indirect risk factor for endometrial cancer [76].

The uterine cavity has long been assumed to be sterile [77]. Later, this hypothesis was challenged after several studies documented the presence of the microbiota uterine by 16S rRNA sequencing [78,79,80,81]. The presence of bacteria in the uterine cavity can occur in three ways: through blood, ascension through the cervix during the follicular and luteal phases of the cycle, and gynecologic procedures like assisted reproductive technology (ART) [8].

The microbiota of the upper genital tract differs from the vaginal microbiota. It is characterized by a low number of bacteria, but it has a greater biodiversity, while the vaginal microbiota has a predominance of *Lactobacilli* [82].

Chen et al. have shown that there is a substantial difference in the microbiota as one moves the female reproductive tract and changes during different phases of the menstrual cycle [82].

In detail, the secretory phase appears to be associated with an increase in bacteria, in particular, *P. acnes*, and an increase in the metabolism of pyrimidines and purines, aminoacyl-tRNAs, and amino acids, and the biosynthesis of peptidoglycan [83].

**Figure 2:**
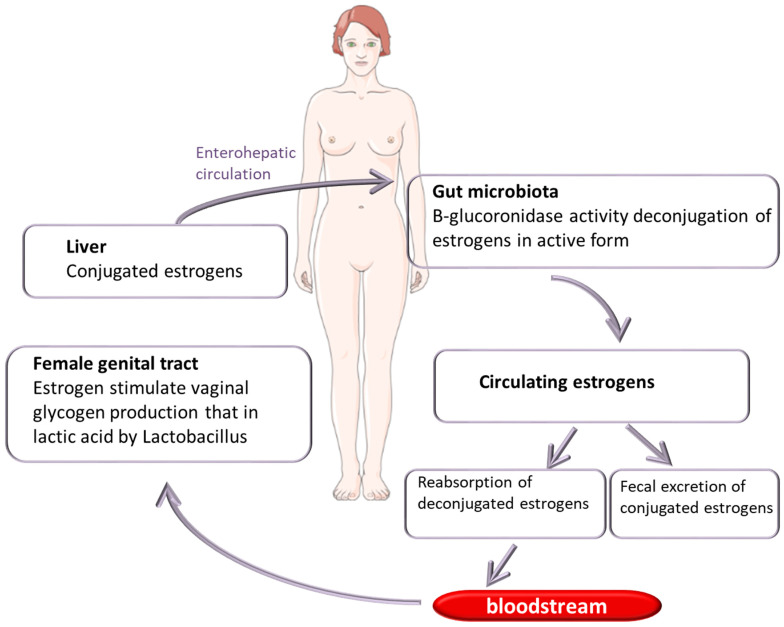
Enteric bacterial genes (estrobolome) produce beta-glucuronidase, an enzyme that deconjugates estrogens in their active form, making them available and free to bind to estrogen receptors and, therefore, able to influence estrogen-dependent processes. An alteration in the estrobolome (dysbiosis) and its regulatory functions leads to an imbalance of various biological processes and an estrogen increase, contributing to the early stages of endometrial cancer.

The presence of specific bacteria, such as *Firmicutes*, *Spirochaetes*, *Actinobacteria* (*Atopobium*), and *Proteobacteria (Bacteroides* and *Porphyromonas*), combined with a high vaginal pH were identified in women with endometrial cancer [84]. *Atopobium* and *Porphyromonas* induce the release of proinflammatory cytokine IL-1α, IL-1β, IL-17α, and TNFα [85]. Interestingly, many tumors, including endometrial cancers, are associated with IL-1α and IL-1β upregulation. IL17α was reported to promote the proliferation of endometrial cells and to contribute to endometriosis due to the induction of proinflammatory and angiogenic factors [86]. Recent studies suggest that the gut–brain axis plays a crucial role in regulating circulating estrogen levels (Figure 2). As a whole, bacteria capable of modulating the enterohepatic recirculation of estrogens and thus influencing the circulating levels of these hormones and their excretion is defined as the ‘‘estrobolome” [87]. The set of bacteria that form the estrobolome produce beta-glucuronidase, an enzyme that deconjugates estrogens in their active form (Figure 2), making them available and free to bind to estrogen receptors and, therefore, able to influence estrogen-dependent processes [88]. An alteration in the estrobolome (dysbiosis) and its regulatory functions leads to an imbalance of various biological processes, promoting the onset of pathologies such as cancer. Wang et al. [89] observed a different microbiota in endometrial cancer (EC) tissues as compared to pericancer tissues. In particular, EC tissues were enriched in the genera *Prevotella*, *Atopobium*, *Peptostreptococcus*, *Anaerococcus*, *Fastidiosipila*, *Finegoldia*, *DNF00809*, *Dialister*, *Peptoniphilus*, *Porphyromonas*, *Anaeroglobus*, and *Criibacterium*, while an abundance of *Lactobacillus* is found in PC tissues [89]. Furthermore, a high abundance of *Prevotella* and high levels of D-dimer were observed in EC tissues, often correlating with disease severity and poor prognosis [90]. Regarding the increase in bacterial population during or after the development of endometrial cancer, potential mechanisms underlying this phenomenon, such as alterations in the local microenvironment favoring bacterial proliferation, compromised host immune surveillance, or changes in bacterial adherence and colonization properties.

These results support the potential role of the microbiota in the manifestation, etiology, or progression of endometrial cancer, which should be further studied.

## 5. Microbiota and Ovarian Cancer

The intersection of microbiota research and ovarian cancer (OC) has emerged as a captivating area of study, shedding light on the potential influence of microbial communities on the initiation and progression of this gynecologic malignancy. Variability in individual microbiota, combined with the multifactorial nature of ovarian cancer, poses challenges in establishing clear causative links. The dynamic interplay between genetics, lifestyle factors, and microbial influences requires further exploration to unravel the complexity of these relationships.

Ovarian cancer represents the second most commonly occurring cancer in women. In 2020, more than 313,000 new cases of ovarian cancer were diagnosed, with 152,000 deaths worldwide, according to worldwide statistics [91]. The unfavorable prognosis is linked to the absence of specific symptoms and signs that allow an early diagnosis, resulting in approximately 70% of cases being diagnosed in an advanced stage of the disease [92]. Ovarian cancer incidence is higher in the postmenopausal age [93]. Research demonstrated that several factors increase the risk of ovarian cancer.

**Endocrine factors:** The risk of ovarian cancer correlates directly with the ovulatory age of the woman; therefore, nulliparity, late menopause, and early menarche represent risk factors. Therefore, pregnancy and the use of oral contraceptives can exert a protective effect on ovarian carcinogenesis by reducing the number of ovulatory events [94]. These oral contraceptives contain a progestin component, which has an apoptotic effect on the ovarian epithelium that is mediated by a modulation of the expression of the isoforms of transforming growth factor (TGF)-β [95].

**Environmental factors:** Ovarian cancer has a greater incidence in industrialized countries, and this seems to be correlated with the typical lifestyle of these countries. In fact, the data in the literature indicate that a diet rich in animal fats and poor in fish and vegetables is a risk factor [96,97,98]. On the other hand, a diet rich in vegetables is associated with lower risk [99].

**Genetic–familial factors:** Although ovarian cancer mostly presents as a sporadic pathology (90–95%), in 10% of cases, a strong familial component has been observed [100]. In fact, the risk of developing ovarian cancer in women who have an affected first-degree relative (mother or sister) is 5% compared to the female population [101]. The genes involved in this type of tumor are BRCA1 and BRCA2. Subjects carrying these mutations have a higher risk of developing ovarian cancer, which is different depending on the gene involved. Women with mutations in the BRCA1 gene have a risk of 20–40% of developing OC, and the risk is 10–20% for women who have a BRCA2 mutation [102]. Recent studies confirmed the existence of a specific ovarian microbiota.

In the cancer samples, *Proteobacteria* and *Firmicutes* were predominant compared to control samples [103]. *Proteobacteria* and *Firmicutes* release bacterial toxins such as colibactin and cytolethal distending toxins, causing cellular DNA damage through double-stranded breaks and thereby activating the DNA-damage checkpoint pathway [104].

Nené et al. 2019 [105] have highlighted that in women with ovarian cancer, there is a depletion of *Lactobacillus* spp. compared to controls. In particular, depletion of *Lactobacillus* spp. is more elevated in patients with BRCA (1/2) mutation; in fact, these mutations seem to be enhancing the growth of community state type non-dominated by *Lactobacillus* spp. [104]. Vaginal lactobacilli utilize vaginal glycogens for the production of lactic acid. High estrogen levels result in the release of glycogens that are produced by vaginal epithelial cells [106].

The glycogen is cleaved by the vaginal α-amylase enzyme into maltose, maltotriose, and α-dextrins, which produce lactic acid through fermentation [107]. Widschwendter et al. [108] highlighted that progesterone levels were higher throughout the menstrual cycle (in particular during the luteal phase) in women with BRCA-mutation carriers compared to controls [108].

**Figure 3:**
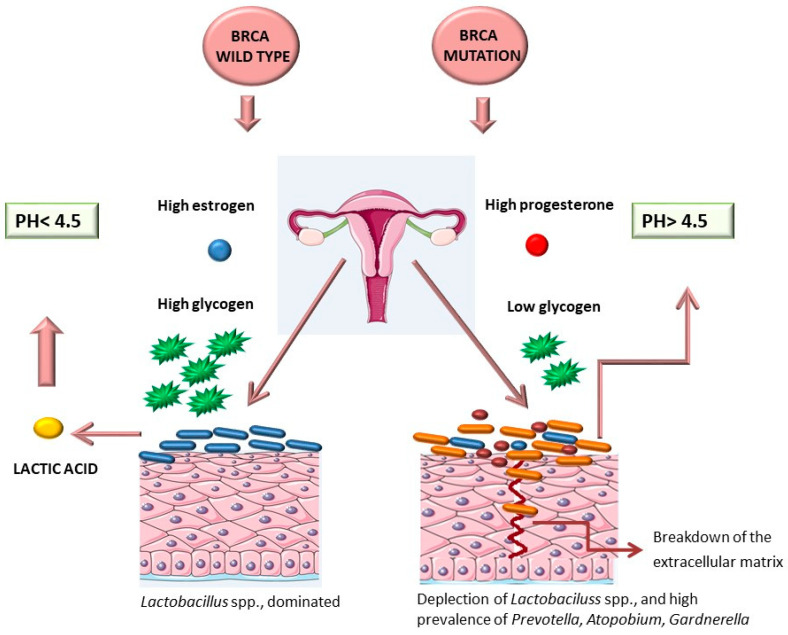
Women with BRCA1 or BRCA2 genes mutation carriers express increased concentrations of progesterone compared to women with BRCA gene wild type, which reduces vaginal glycogen concentrations, resulting in an environment that is less favorable to the growth of Lactobacilli. High estrogen levels in BRCA wild type release glycogen from vaginal epithelial cells that is cleaved by the vaginal α-amylase enzyme into maltose, maltotriose, and α-dextrins, producing lactic acid through fermentation. Lactic acid keeps the vaginal pH around 4.5, inhibiting pathogen invasion and growth.

High levels of progesterone concentrations reduce vaginal glycogen concentrations, triggering an environment that is less favorable to the growth of community-type lactobacilli [105] (Figure 3). In a recent study, the antibodies Pgp3 and CHSP60-1 against *C. trachomatis* were found to be associated with an increased risk of ovarian cancer through an antiapoptotic effect, which in turn favors the survival of DNA-damaged cells, leading to an increased risk of cancer onset [109].

Recent evidence has shown that during tumorigenesis, due to mucosal destruction, some microorganisms may invade the tumor from adjacent normal sites, giving rise to “intratumoral microbiota” [110]. The latter contributes to the promotion, initiation, and progression of cancers by DNA mutations, activating carcinogenic pathways, promoting chronic inflammation, and activating the complement system, initiating metastasis [110].

In addition, intratumoral *Propionibacterium acnes* found in ovarian cancer tissue seems to play a key role in cancer progression by aberrant hedgehog signaling activation [111]. In vitro studies have demonstrated that the original bacterial molecule, such as lipopolysaccharide *Escherichia coli*, can induce the release of pro-inflammatory cytokines in ovarian cancer cells, promoting tumor growth and chemo-resistance through the stimulation of TLR (toll-like receptor) receptors [112].

Recent investigations have revealed a possible correlation between the gut microbiota and ovarian cancer. Evidence suggests that the gut microbiota plays a role in modulating systemic inflammation and immune responses, thereby affecting the ovarian tumor microenvironment. Dysbiosis, characterized by an imbalance in microbial composition, has been implicated in creating an inflammatory milieu conducive to cancer development [113].

In particular, in ovarian cancer samples, specific bacterial Firmicutes have been identified, such as *Abiotrophia*, *Bacillus*, *Enterococcus*, *Erysipelothrix*, *Geobacillus*, *Lactobacillus*, *Lactococcus*, *Listeria*, *Pediococcus*, *Peptoniphilus*, and *Staphylococcus* [102].

Moreover, intestinal dysbiosis can promote the epithelial–mesenchymal transition (EMT) and the growth of xenograft tumors in mouse models through the activation of macrophages and, consequently, the production of tumor necrosis factor-alpha (TNF-α) and interleukin-6 (IL-6) in the peripheral blood [114]. The gut microbiota plays a central role in understanding the mechanism of cancer development and may influence current anticancer strategies.

## 6. Diagnosis, Treatments, and Future Therapeutic Strategies

The bacterial signatures present in the gut and the female genital tract may have clinical implications. Several studies have highlighted that specific microbiota can be an effective tool both as a diagnostic biomarker and for targeted therapeutic strategies for gynecological cancers. The future aim is to explore personalized therapeutic approaches based on an individual’s unique microbiota profile. As previously discussed, several factors influence the composition of the microbiota, such as environmental factors, age, hormonal factors, lifestyle, and especially nutrition. Diet plays a fundamental role in modulating our intestinal microbiota. A healthy and balanced microbiota is maintained, above all, thanks to the intake of prebiotics and probiotics [115]. In particular, foods rich in fiber produce an increase in microbial diversity; the bacteria produce metabolites, the most important being SCFAs (short-chain fatty acids), which bring benefits and positive effects to human health as they participate in the restoration of intestinal balance, nutrient metabolism and protection of the immune system [116]. On the contrary, a diet based on the high consumption of processed red meat and refined sugars would tend to cause intestinal imbalance, increase the inflammatory state, and cause the onset of diseases [117]. Characterization of the vaginal microbiota will therefore pave the way for new integrative therapies based on probiotics, antibiotics, and vaginal microbiota transplantation (VMT) that can shape the composition of the female reproductive tract (FRT) microbiota and restore eubiosis. The administration of probiotics can restore the normal balance of the vaginal microflora, contributing to the well-being of the female genital tract. Vaginal probiotics are predominantly made up of *Lactobacillus* spp. and can be administered via capsules or swabs for local application, or they can be taken orally in a format that allows their survival along the gastrointestinal tract and allows bacteria to pass from the intestine to the vaginal tissue and colonize it. Researchers propose an antitumor effect of probiotics in the colon through several mechanisms: suppression of pro-carcinogens and carcinogens, activation of the immune system of the host, modification of the transit time and motility of the colon, inhibition of the bacteria involved in the transformation of pro-carcinogens to carcinogens or reducing the intestinal pH [118]. Similarly, in cervical cancer, probiotics have shown several anticancer effects, including suppression of cell viability, inhibition of metastasis, reduction in proliferation, and induction of apoptosis [119,120,121]. Wang et al. [122] demonstrated that *L. Crispatus*, *L. jensenii*, and *L. gasseri* supernatants impede the proliferation of Caski cells, leading to an increase in the number of cells in the S phase and, conversely, a significant decrease in the number of cells in the G2/M phase. Furthermore, the use of *Lactobacillus* spp. supernatants caused a reduction in the expression of the oncogenes CDK2, cyclin A, and the E6–E7 genes [121]. Several pieces of evidence suggest that antibiotic treatment has a strong impact on microbiota composition. The literature suggests that in vitro antibiotic treatment may block cell proliferation, reduce the number of stem cells in ovarian cancer, induce apoptosis, and inhibit the EMT [123,124,125,126]. Moreover, the use of antibiotics in combination with drugs has been used to act directly on tumor cells in in vitro models to improve the effect of chemotherapy. The therapies currently available for gynecological cancers include surgery, radiotherapy, chemotherapy, and immunotherapy, including antiangiogenic factors targeting VEGFR. Several studies suggest that the microbiota may interact with pathways targeted by standard-of-care (SOC) therapies, potentially influencing treatment outcomes in various tumor types through a number of mechanisms, including enzymatic degradation, translocation, and immunomodulation [17]. The influence of the microbiota and the effectiveness of therapies has been observed in various types of tumors, but it is not yet entirely clear whether the vaginal microbiota plays a role in influencing the effectiveness of therapies.

To date, few research studies investigated the link between vaginal microbiota and anticancer therapies in the frame of gynecological cancers. Tsementzi et al. [127] reported that pelvic radiation negatively affects vaginal microbiota, with several symptoms rising after one year accompanied by an enrichment of opportunistic pathogens [127].

In support of this, it has been observed that the administration of *Lactobacillus delbrueckii* subsp. *lactis* in women with gynecological cancer, it reduced symptoms and dysbiosis caused by radiotherapy [128]. Therefore, it is important to explore whether the vaginal microbiota plays a role in therapeutic lines, as this could impact treatment response and disease progression in gynecological cancers. These questions highlight important areas for future research to advance our understanding of the role of the microbiota in gynecological cancers.

However, in order to understand the role of antibiotics in cancer, in vivo studies are needed. Another potential approach consists of vaginal microbiota transplantation (VMT) in order to treat endometriosis, other than in the management of gynecologic cancer and its post-therapeutic complications [129]. VMT is a technique that involves grafting the entire vaginal microbiota deriving from a healthy donor to the receptor having vaginal microbiota dysbiosis [130]. Firstly, donors are recruited and screened; later, cervicovaginal fluid (CVF) samples are collected from healthy donors and processed. Finally, healthy vaginal microbiota are transplanted into the vagina of the dysbiotic patients in order to restore the healthy vaginal microbiota with the intent to rescue normal function [131]. In several studies, VMT has been successfully used. In women suffering from bacterial vaginosis, VMT has been used to restore the microbiota and reduce symptoms [130]. In this study, has been observed a restoration of vaginal flora dominated by lactobacilli and long-term relief of symptoms [131]. In murine models after VMT, inhibition of the progression of endometriotic lesions, a reduction in inflammatory cytokines, and the downregulation of key proteins of the NF-κB signaling pathway were observed [130]. More detailed studies on the effectiveness of VMT in treating all vaginal disorders are needed, as at present VMT is currently limited to the treatment of bacterial vaginosis [131]. However, the main limitations of this approach are related to safety problems and the transmission of unknown pathogenic bacteria [132]. Similarly, the Food and Drug Administration (FDA) has warned about the potential risk of the use of fecal microbiota transplantation after six patients who received FMT from a stool bank company based in the United States developed infections. In particular, these infections were due to the presence of enteropathogenic *Escherichia coli* (EPEC) and Shigatoxin-producing *Escherichia coli* (STEC) since the stool specimens were not screened before use for ESBL-producing Gram-negative organisms [133]. Moreover, in 2019, cases of multidrug-resistant organism (MDRO) infections attributed to FMT have been reported, one of which led to the patient’s death [134]. In addition, a patient, after receiving feces from a healthy but overweight donor, developed obesity [135]. Therefore, the FDA recommends thorough screening of donors, although some unknown pathogens may escape common screening tests and be transmitted to patients. However, as stated above, personalized therapeutic approaches based on an individual’s unique microbial profile may represent an important potential for cancer treatment, although they have limitations.

Personalized medicine often involves expensive molecular profiling and targeted therapies, which may not be accessible to all patients or all healthcare systems, and requires sophisticated diagnostic and interpretative techniques, which can be complex and lead to delays in starting treatment. Additionally, tumors can develop resistance to targeted therapies over time, leading to treatment failure and disease progression. Moreover, personalized treatments may not address the heterogeneity of tumor cells within a tumor, increasing the risk of recurrence [136]. Successful implementation of personalized medicine requires collaboration across multiple healthcare disciplines, including oncologists, geneticists, pathologists, and bioinformaticians, which can be logistically challenging.

## 7. Conclusions

The mechanisms according to which the microbiota are involved in the development and progression of cancer are not yet fully understood, and whether an altered microbiota may be more the effect than the cause. However, several studies demonstrate that vaginal dysbiosis and the presence of pathogens that release toxins and express virulence factors can induce inflammation through the release of pro-inflammatory factors, production of carcinogenic metabolites, and modulation of host immune responses. All these factors are involved in the pathogenesis and progression of gynecological cancer. Furthermore, there is a close relationship between the intestinal microbiota and circulating levels of estrogen (estrobolome), the alteration of which can lead to the onset of estrogen-dependent gynecological tumors such as ovarian and endometrial cancer. However, the possibility that some bacteria are not detected in routine screening still represents a limitation. Routine screening methods may have detection thresholds that limit their ability to identify low-abundance or fastidious bacterial species present in the microbiota. Additionally, sampling methods used in routine screening, such as swabs or biopsies, may not capture the full diversity of bacteria present in a particular body site, leading to potential underrepresentation of some species. The composition of the microbiota can vary over time due to factors such as diet, drug use, and environmental exposures. Addressing these limitations may require the development of more sensitive and comprehensive screening techniques, as well as considering the integration of multi-omics approaches to provide a more holistic view of the microbiota and its role in health and disease. Further studies are needed to better understand the multifaceted interlink between the microbiota and gynecologic cancers to identify microbial signatures and provide a glimpse into future personalized interventions that can redefine the prevention and treatment landscape.
